# Do Functional Keratin Dressings Accelerate Epithelialization in Human Partial Thickness Wounds? A Randomized Controlled Trial on Skin Graft Donor Sites

**Published:** 2013-08-29

**Authors:** Andrew Davidson, N. Hamesh Jina, Clive Marsh, Martin Than, Jeremy W. Simcock

**Affiliations:** ^a^Departments of Plastic and Reconstructive Surgery, Christchurch, New Zealand; ^b^Departments of Emergency Medicine, Christchurch Hospital, Christchurch, New Zealand; ^c^Departments of Keraplast Research LLC, Christchurch, New Zealand

**Keywords:** acute wounds, donor site, epithelialization, keratin, partial thickness wounds

## Abstract

**Objective:** To determine if the experimental (keratin-based) dressing accelerates epithelialization rates during healing of partial-thickness wounds, relative to a Standard Care dressing. **Method:** A randomized control trial was conducted using a Standard Care dressing side by side with the experimental dressing on a sample (n=26) of partial-thickness donor site wounds. The proximal/distal placement of the control and treatment was randomized. Percentage epithelialization after approximately 7 days was estimated from which time to fully epithelialize can be inferred. Patients were grouped into “young” (≤50 y/o) and “old” (>50 y/o). **Results:** For the “old” patients (n=15), the median epithelialization percentage at 7 days is 5% and was significantly (*P*=.023) greater for the experimental dressing. For the “young” patients (n=11), the median epithelialization percentage at 7 days was 80% and there is no significant difference between the experimental and Standard Care control dressings. **Conclusions:** The experimental dressing significantly increases the rate of epithelialization of acute, traumatic partial-thickness wounds in older patients. We suggest that the dressing may be clinically useful in similar situations where epithelialization may be delayed because of patient or wound characteristics.

Keratinocyte proliferation and migration plays a key role in the reepithelialization of cutaneous wounds. Upregulation of various intermediate filament genes such as Keratins 6, 16, and 17^1^ is central to this process. Apart from being important to the structural integrity of skin, these inducible keratins regulate cell growth and migration via the Akt/mTOR signalling pathway.^2^ It is thought that the activation of these genes therefore is integral to effective cutaneous wound healing, and Keratin 16 has been shown to be the most down-regulated gene in the bed of nonhealing ulcers when compared with healing wounds.^3^ The critical role of Keratin 17 has similarly been shown as the absence of the gene results in markedly impaired healing.^2^ Research into exogenous keratins is showing promise in the treatment of cutaneous wounds by stimulating keratinocyte activation, inducing the expression of endogenous keratins, and accelerating reepithelialization.

A new range of keratin-based dressings (Replicine™ by Keraplast, San Antonio, Texas [www.keraplast.com]) have demonstrated the ability to upregulate the migration and proliferation of keratinocyte cells.^4^ The constituent keratin protein is (so-called) “Functional” because it retains its structural form and is able to perform its biological function. The dressings have further demonstrated that they can accelerate epithelialization rates of acute, deep partial-thickness wounds in an in-vivo porcine model study^5^ and acute wounds in Epidermolysis Bullosa patients^6^^,^^7^ and in skin-tear injuries.^8^ Further clinical studies conducted on chronic wounds (venous leg ulcers) have also observed faster healing rates.^9^^,^^10^

Split skin graft donor sites have been shown to respond to moist healing,^11^ but a comprehensive meta-analysis^12^ did not demonstrate an effect of other factors. Various exogenous growth factors have been investigated and believed to have potential but have not been adopted in standard clinical practice.^13^^,^^14^

We aimed to compare epithelization rates achieved by keratin-based dressings with standard wound care for partial-thickness acute wounds in humans in a clinical setting.

## METHODS

### Setting and participants

We enrolled consenting adult patients undergoing a split skin graft as part of reconstructive surgery. The split skin graft donor site was the thigh in all cases except one where it was taken from the medial arm. The thickness of skin graft harvested was determined by the reconstructive need (typically 10/1000-in thick).

### Study design

This was a randomized controlled trial in which part of the graft donor site was used as an internal control for each individual patient. The donor site wound was dressed half with a control dressing and half with an experimental (keratin) dressing. The proximal or distal location of each dressing type (control or experimental) was determined using sequential prerandomized, sealed, opaque envelopes. Split skin graft donor site wounds were chosen for this study, because they provide uniform thickness wounds for making epithelialization rate comparisons. In addition, they are sufficiently large to allow Treatment and Control dressings to be used side by side to provide an internal control for each patient.

### Control dressing

This was an alginate dressing (Algisite [Smith and Nephew, London]), applied postsurgery, and left in-situ for 2 weeks (at which time complete healing is expected to have occurred). This was standard care for donor sites at our facility.

### Experimental dressing

Keramatrix® (Keraplast, San Antonio, TX) is one of the dressings in the Replicine™ keratin dressing range. This was chosen as the Treatment for the study because its exudate handling properties and longevity matched the needs of a skin graft donor site.

### Outcome measurement

The primary outcome was the extent (percentage) of epithelialization in proximal and distal thirds of the wound at 7 days after surgery. The middle third of the wound was ignored as it potentially may have been influenced by both dressings. The percentage epithelialization was estimated by an experienced clinician, blinded to treatment allocation who made visual assessments in controlled lighting conditions in clinic. Photographs were taken for reference but do not reliably discriminate neoepithelium from sheen of wound moisture.

Secondary assessment was by a questionnaire completed by outpatient department nurses at the time of 7-day follow-up. They were asked to assess each half of the dressing for ease of removal and pain on removal.

### Statistical methods

A 2-tailed, paired, *t*-test approach was used to determine if there were significant differences between the percentage epithelization levels of the Treatment and Control portions of each donor site. Patients older than 50 years and those 50 years old or younger were analyzed as separate groups.

The study was approved by the institutional review board (Upper South B Regional Ethics Committee, New Zealand). The approved study protocol described the data and subgroup analysis plan.

## RESULTS

Thirty-seven patients were enrolled into the study. There were no adverse events relating to either Treatment or Control dressing. Eleven patients were not able to be assessed on postoperative day 7 leaving 26 participants available for analysis, 15 were older than 50 years (median age=73 years) and 11 were 50 years old or younger (median age=35). Reasons for not completing assessment were as follows: did not attend follow-up clinic (5), attended clinic too late to be included in assessment (2), dressings changed early by on-ward nursing staff (2), and incomplete consent process (2).

**Figure 1:**
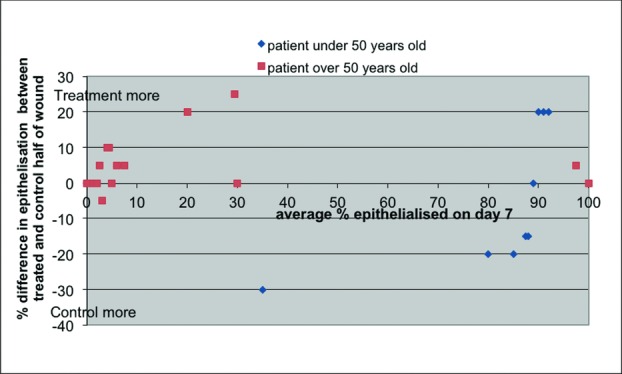
Difference in epithelialization of adjacent sides of donor site wound Treatment vs Control.

Percentage of epithelization varied markedly across the patients overall. This difference was predominantly associated with age (see Table 1 and Fig 1). In the older (>50 years) group, the majority of each wound was unhealed (median epithelialization percentage, 5%). In this group, there was a significant (*P*=.023) difference between the epithelialization in the Control portion and the Treatment portion of the wound. The corresponding medians of the Control group and Treatment group were 5% and 10% epithelialization, respectively.

In the younger (≤50 years) group, epithelization was almost complete at 7 days (median epithelialization percentage, 80%). There was no significant difference between Treatment and Control portions of the wound.

**Figure 2:**
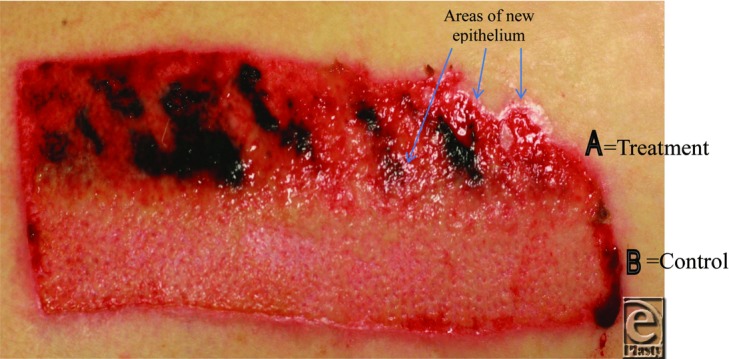
Photograph showing epithelialization of a typical donor site 7 days after surgery.

Figure 1 shows the difference in epithelialization, approximately 7 days after surgery, for all patients measured. This illustrates the difference between the responses of older (>50 years) to younger (≤50 years) patients. Figure 2 shows a typical outcome for the older group: side “A” (Treatment) shows early epithelialization from the epidermal appendages (5% epithelialization) whereas side “B” (Control) has not started to epithelize (0% epithelialization). The Treatment side demonstrates coagulated blood on the surface of the wound as the Treatment dressing is not designed to be hemostatic (unlike the Control dressing). The wound was gently washed with normal saline and adherent slough or blood indicated that the region had not epithelialized; this was taken into account by the blinded assessor.

Qualitatively, the Treatment dressing was observed to handle the high exudate load during the first ˜2 days from the donor sites well. Sixteen of 27 patients followed up had questionnaires completed by nurses of which 6 felt that the side-allocated Treatment dressing was less painful to remove, one found the Control dressing side less painful, and the rest noted no difference. Subjectively nurses typically commented that the Treatment dressings were easier, less painful, and less traumatic to remove than the Algisite.

## DISCUSSION

Older patients healed their partial-thickness wounds more slowly; however, there was significantly more epithelialization after 7 days in wound portions treated with the keratin-based dressing compared with standard care. This implies that the time to complete epithelialization may be reduced in the portion of the wound treated with the keratin-based dressing, for the older patients. This study also demonstrated rapid epithelialization of split skin graft donor sites in young patients. This is consistent with previous reports^15^ and reflects ideal wound healing. There was no significant difference in epithelization rates between Treatment and Control dressings for younger patients.

Delayed epithelialization is commonly caused by both patient factors (age and comorbidities) and wound factors (deep partial-thickness injuries including deep dermal burns) and is associated with wound complications such as infection and scarring.^16^ Thus, mitigation of delayed epithelialization is of clinical significance and the results suggest that keratin-based dressings can achieve this in some cases. The clinical implications of this study are that keratin-based dressings can reduce the delayed epithelialization seen in predisposed patients and so reduce the complications of healing in this group of patients. The results may extrapolate from these traumatic, acute partial-thickness wounds to thermal, acute partial-thickness wounds such as burns.

The results of this study are consistent with the proposed mechanism of action and with earlier published preclinical results and other clinical studies. The proposed mechanism, as described in the “Introduction,” is that the exogenous keratin can stimulate keratinocytes via the Akt/mTOR signalling pathway. The clinical observations in this study are consistent with such stimulated keratinocyte activity. However, ethical considerations did not permit biopsy to permit investigation of the response to the Treatment at a cellular level. Furthermore, both preclinical animal studies^5^ and other clinical studies^6^^-^8 showed similar increases in epithelialization rates and are consistent with the findings of this study.

Questionnaires, completed by nurses, indicated that the Treatment dressing was well tolerated by patients, and ease of removal was at least comparable to current standard care. Although there was more coagulated blood on the surface of the Treatment side of wounds, this does not seem to have adversely affected the ease of dressing removal or the rate of epithelialization and healing.

Potential criticism of this study includes the small sample size, especially given the heterogeneity of this patient population. However, the use of internal controls meant that we were able to detect significant difference between Treatment and Control dressings. The wide range of epithelialization rates observed in this study supports using internal controls in the investigation of partial-thickness wound healing rates. The method of assessment, estimation of percentage of epithelialization by a skilled, blinded assessor, is novel. As expected, the neoepithelium was observed to be in patches around the epidermal appendages and the visual assessment method was found to be appropriate to estimate the percentage epithelialization. A weakness of the assessment technique is that it is difficult to repeat, the skilled assessor was confident in his ability to assess “in the flesh” but the digital images were unable to capture detail necessary to make the assessments of percentage epithelialization and hence assessments from the digital images are not reproducible.

Ideally, time to complete epithelialization could be measured for each patient, but a method that observes the wounds daily as they near complete epithelialization is onerous and disruptive for patient's healing. Thus, this method of a single observation at 7 days was used. We suggest that there will be a strong, direct relationship between the percentage epithelialization after 7 days and time to complete epithelialization.

## CONCLUSIONS

Keratin-based dressings significantly increase the rate of epithelialization of acute, traumatic partial-thickness wounds in older patients. We suggest that they may be clinically useful in similar situations where epithelialization may be delayed because of patient or wound characteristics.

## Figures and Tables

**Table 1:** Extent of epithelialisation for each patient

Patient number	Epithelialization for treatment, %	Epithelialization for control, %	Difference in epithelialization (Treatment − Control), %
Patients >50 years old			
1	30	30	0
2	42	17	15
3	10	0	10
4	100	100	0
5	10	5	5
6	10	5	5
7	5	5	0
8	5	0	5
9	0	0	0
10	10	0	10
11	0	5	−5
12	100	95	5
13	30	10	20
14	0	0	0
15	0	0	0
Patients ≤50 years old			
1	100	80	20
2	90	90	0
3	0	0	0
4	100	80	20
5	75	95	−20
6	0	0	0
7	80	95	−15
8	80	95	−15
9	20	50	−30
10	70	90	−20
11	100	80	20
